# Therapeutic drug monitoring of meropenem and piperacilin administered in continuous infusion. preliminary data

**DOI:** 10.1186/2197-425X-3-S1-A394

**Published:** 2015-10-01

**Authors:** M Miralbés Torner, F Ahmad Diaz, S Carvalho Brugger, SM Cano Marron, JA Schoenenberger, A Aragones Eroles, X Nuvials Casals, M Vallverdu Vidal, B Balsera Garrido, M Palomar Martinez

**Affiliations:** Hospital Universitari Arnau de Vilanova, Lleida, Spain; Hospital Universitari Arnau de Vilanova. IRB Lleida, Lleida, Spain

## Introduction

Piperacilin-tazobactam (TZP) and meropenem (MER) are two extended-spectrum beta-lactams antibiotics (ATB) broadly used in intensive care units. We know that early and appropriate ATB treatment remains the most important intervention in septic patients, so optimization of ATB administration should be a priority.

## Objectives

Evaluate the effectiveness of MER and TZP administered in continuous infusion in critically ill patients in order to maintain concentrations 4-6 times above the minimum inhibitory concentration (MIC) for 100% of the interval time of infusion.

## Methods

Open, prospective, single-center study. All consecutive patients in whom treatment with MER or TZP was indicated from October 2014 to March 2015 were included. A 2g (MER) or 4g (TZP) loading dose was given followed by a 6g (MER) or 16-24g (TZP) continuous infusion over 24 hours. Serum concentrations were determined by high-performance liquid chromatography (HLPC) 1 hour, 24 hours and 3-5 days after the start of the infusion, determining maximum (Cmax) and free steady state concentrations (ƒCss). The objective was maintaining ƒCss 4-6 times above the MIC corresponding to the clinical breakpoint for *Pseudomonas aeruginosa* from our hospital database: 8 µg/ml for MER and 16/4 µg/ml for TZP. When the target was not achieved, the dose was adjusted.

## Results

We enrolled 49 patients (73% male and 27% female) and determined 60 Css. Mean APACHE-II score was 16 ± 7. Empiric therapy was administered in 48 cases (80%). 13 patients (26%) were admitted to ICU after ≥ 7 days of hospitalization. 25 patients (51%) had septic shock, 2 (4%) severe sepsis and 1 (43%) sepsis. 1 patient (2%) had not systemic inflammatory response (SIR). 9 patients (18%) had bacteremia. We analyzed Css of MER/TZP during the first 24-48 hours, which are shown in Table1. Css determination led to the dose titration in 30% of the treatments.

**Table 1:** Css within the first 24-48 hours.

antibiotic	Cases	ƒCss < 4MIC	ƒCss 4-10 MIC	ƒCss >10 IMC
Meropenem 2 g	14	6 (43%)	7 (50%)	1 (7%)
Piperacilin-tazobactam 16g	15	2 (13 %)	9 (60%)	4 (27%)
Piperacilin-tazobactam 24g	31	11 (35%)	16 (52%)	4 (13%)
Total	60	19 (32%)	32 (53%)	9 (15%)

## Conclusions

Although ATB optimization, 32% of patients did not achieve our outcome. Therapeutic drug monitoring allows us to adjust dose administrated based on Css in order to achieve optimal drug exposure for an individual patient. Based on our results, dosages needed in critically ill patients might be markedly higher than those currently administered. We remark the need to keep analyzing cases to assess risk factors for ATB underdosing and continue monitoring Css to evaluate further dose adjustments.

## Grants

Grant of the Insitut de Recerca Biomédica de Lleida (IRB)

## References

[1] Roberts JA (2010). Therapeutic drug monitoring of β-lactams in critically ill patients: proof of concept. Int J Antimicrob Agents.

